# A cross-sectional study on sleep disturbances and associated factors among nurses

**DOI:** 10.1186/s12888-022-03748-y

**Published:** 2022-02-15

**Authors:** Tuan Van Nguyen, Hsueh-Erh Liu

**Affiliations:** 1 Faculty of Nursing and Medical Technology, Can Tho University of Medicine and Pharmacy, Can Tho, Vietnam; 2 School of Nursing, College of Medicine, Chang Gung University, No.259, Wenhua 1st Rd., Guishan Dist, Taoyuan City, 33302 Taiwan, ROC; 3 Department of Rheumatology, Chang Gung Memorial Hospital, Linkou, Taoyuan, Taiwan, ROC; 4 Department of Nursing, College of Nursing, Chang Gung University of Science and Technology, Taoyuan, Taiwan, ROC

**Keywords:** Nurses, Risk factors, Sleep disturbance, Sleep quality, Vietnam

## Abstract

**Background:**

Many studies have measured sleep disturbances among nurses globally, but none have addressed this issue in Vietnam. Therefore, this study aimed to assess the prevalence of sleep disturbance and identify associated factors among staff nurses in Vietnam.

To identify sleep disturbances and associated factors among staff nurses in Vietnam.

**Methods:**

A cross-sectional design was used in this study. Participant nurses (*n* = 420) completed a general information questionnaire, the Vietnamese version of the General Sleep Disturbance Scale (GSDS), the Perceived Stress Scale, and the Short Form 12. Data were collected between July and September 2019 from three public hospitals located in southwestern Vietnam. Data were analyzed using Chi-square, independent Student’s t-test, and multivariate logistic regression analysis.

**Results:**

The average GSDS score was 41.10 (SD = 19.48), indicating sleep disturbances among 46.9% of nurses. Age, health condition, stress, and quality of life had an impact on sleep disturbance. Multivariate logistic regression analysis confirmed that nurses with high stress and poor physical health status were more likely to experience sleep disturbances.

**Conclusion:**

Vietnamese nursing staff suffers from a high rate of sleep disturbances. Significant predictors included high stress and poor physical health status. Administrators of healthcare services should carefully consider how to conduct interventions to help the nurses handle their sleep disturbances such as stress management and physical health promotion.

## Background

Sleep disturbances are a common health issue worldwide. It includes many sleep complaints, such as trouble initiating or maintaining sleep, early morning awakening, short sleep duration, excessive daytime sleepiness, etc., [1–4]. Sleep disturbances are understood as physical and psychological states that cause a series of adverse effects because of the abnormal amount and poor quality of sleep (QoS) [5];. Epidemiological studies have shown that the prevalence of worldwide sleep disturbance in the general population ranges from 8.3 to 45% [2]. Approximately 20% of people experience sleep disturbances in America [6], 10% in western industrialized countries [7], and 26.4–39.4% in Asia [8].

Nurses work as special occupational groups. They face a relatively high number of physical and mental challenges every day, including busy work schedules, many job duties to patient care, and regular exposure to disease and death [9, 10]. Moreover, nurses are asked to provide continuous healthcare, so they often have rotating shift work [4, 11]. In particular, the shortage of nurses is becoming a worldwide healthcare issue. This shortage has led to an increased demand for nursing care. Thus, health facilities may require nurses to work extended shifts and run more shifts than necessary [12–14]. These working mechanisms disrupt natural circadian rhythms that can lead to loss of quantity and QoS, resulting in extended sleep impairment [4, 9, 15]. Consequently, nurses are more susceptible to sleep disturbances than the general population and other healthcare workers [2, 4].

Previous literature reveals that the prevalence of sleep disturbances among nurses is high in different countries. In China, 63.9% of nurses reported poor QoS [9]. This number was 56% in Iran [16], 68% in Jordan [17], 79.8% in Korea [18], 57.8% in Malaysia [19], 61% in Nigeria [13], 75.8% in Taiwan [8], 61.9% in Turkey [20], 78% in the UK [21], and 63% in the US [22].

Sleep disturbances among nurses have been reported to be associated with not only their health but also low work performance, low job satisfaction, low quality of life (QoL), medical errors, and even reduced patient safety [4, 5, 10, 11, 18, 23].

Several factors associated with sleep disturbances among nurses have been reported in the literature. Particularly, old age has a negative effect on QoS [20, 24]. Female nurses have a higher risk of experiencing sleep disturbances than male nurses [5, 9, 24]. Nurses with a high school or lower-level degree have a higher risk of experiencing sleep disturbances than those with a college or higher education degree [8]. In particular, shift-working is a strong predictor of sleep disturbance among nurses. An increasing rate of night shifts per week plays a major role in decreasing QoS [9, 16, 20, 24, 25]. Moreover, a high level of work stress plays an important role in increasing sleep disturbances [5, 9]. Furthermore, poor QoL was also found to be closely associated with poor QoS [10, 11].

Vietnam is currently experiencing a nursing shortage, with 11.7 nurses per 10,000 residents. In hospitals, the nurse-to-patient ratio is also extremely low, with one nurse caring for as many as 20 to 30 patients. Nurses work 12-h shifts and occasionally 24-h shifts, which adds to their stress [26]. An investigation of 287 Vietnamese nurses conducted recently discovered that 43.5% of them were stressed out [27]. An association between stress and sleep disturbances has also been established in the literature [17, 28, 29]. In addition, inadequate facilities and substandard working conditions are also noted [26]. The above factors may contribute to increased the prevalence of sleep disturbance among Vietnamese nurses. Although many studies have focused on measuring sleep disturbances among nurses around the world, to the best of our knowledge, no studies addressed this issue in Vietnam. Therefore, this study assessed the experience of sleep disturbance among staff nurses and identifies factors that have an impact on it in the Vietnamese context. Based on the literature, the current study analyzed associations of demographic characteristics (e.g., age, gender, education level, shift work, etc.), stress, and QoL with sleep disturbance. We believe that the findings of this study will contribute significantly to the literature.

## Methods

### Study design

This was a cross-sectional study. The Strengthening the Reporting of Observational Studies in Epidemiology guidelines were applied in this study [30].

### Setting and sampling

A convenience sample of staff nurses (*n* = 420) was recruited from three public hospitals located in the southwestern part of Vietnam. Data were collected between July and September of 2019. The inclusion criteria were as follows: full-time working nurses, 18 years old and above, and willingness to participate in the study. Participants who were absent during data collection, such as on sick leave or delivering a baby, were excluded.

Participants were explained the study’s aims, benefits, and risks, the procedure for ensuring confidentiality, and the voluntary nature of participation. Written informed consent forms were signed immediately after they agreed to participate in this study. Then, the participants were obtained from all participants before beginning the study. Subsequently, participants were required to complete the questionnaires within 20 to 30 min and return them to the data collector.

### Instruments

#### Sample characteristics

Participants responded to question regarding demographic characteristics, including age, gender, marital status, education level, self-rated health conditions, work experience, and shift work.

#### Assessment of sleep disturbance

The General Sleep Disturbance Scale (GSDS) was used to assess the range of sleep issues over the last 7 days [31]. This scale consists of 21 items across seven subscales: problems initiating sleep (1), waking up during sleep (1), waking too early from sleep (1), QoS (3), sleep quantity (2), fatigue, and alertness at work (7), and use of substances to induce sleep (6). Each item was scored using an 8-point Likert scale ranging from 0 (never) to 7 (every day). The overall scores were obtained by reversing the scores on three items (items 4, 10, and 11), and then summing all 21 items. The total possible score for this scale ranged from 0 to 147. A total score of 43 or above represents a general sleep disturbance. For the subscales, an average score of 3 or above represents a specific sleep problem. The overall Cronbach’s alpha was .88 [31]. The Vietnamese version of the GSDS has been translated and reported with Cronbach’s alpha of .81 [32].

#### Assessment of perceived stress

The Perceived Stress Scale (PSS-10) was used to measure participants’ levels of stress [33]. This scale consists of 10 items rated on a 5-point Likert scale (0 = never, 4 = very often). The scale asks how often respondents experienced particular thoughts and feelings during the last month. The overall scores were obtained by reversing the scores on four items (items 4, 5, 7, and 8) and then summing all 10 items. The total scores ranged from 0 to 40, where the level of perceived stress was categorized as low stress (score < 13), moderate stress (score between 14 and 26), and high perceived stress (score > 27). The Cronbach’s alpha of the PSS-10 was .78 [33]. The Vietnamese version of PSS-10 has been translated and reported with Cronbach’s alpha of .8 [32].

#### Assessment of quality of life

The Short Form 12 (SF-12) was used to measure the general physical and mental health of participants [34], across two domains: physical component domain (PCS) and mental component domain (MCS). PCS has six items across four dimensions: general health (1), physical functioning (2), bodily pain (1), and role limitation associated with a physical health problem (2). Similarly, MCS has six items across four dimensions: mental health (2), social functioning (1), vitality (1), and role limitation due to emotional health problems (2). The SF-12 was scored using a standard scoring procedure. Scores on the PCS and MCS ranged from 0 to 100. Higher scores indicate higher levels of physical and mental health. The SF-12 had a Cronbach’s alpha of .79 and had a good test-retest reliability with PCS = 0.89 and MCS = 0.76 [34]. The Vietnamese version of the SF-12 has been translated and reported with Cronbach’s alpha .88 [32].

### Data analysis

Statistical Package for the Social Sciences (SPSS) for Windows (version 23.0; IBM Corp.) was used to analyze the data. The statistical significance level was set at *p* = .05. Mean and standard deviation (*SD*) were used to summarize the continuous variables (e.g., age, work experience, QoL, perceived stress, etc.), and frequency and percentage (%) were used to describe categorical variables (e.g., gender, education level, shift work, sleep disturbance, etc.). Furthermore, the chi-square test for categorical variables and independent Student’s t-test for continuous variables were used to examine the association between sleep disturbance and demographic factors, perceived stress, and QoL. Multivariate logistics regression analysis, with odds ratios (ORs) and 95% confidence intervals (95%CIs) were calculated to identify the predictors of sleep disturbances [35].

## Results

### Demographic information of the participants

The general characteristics and descriptive variables of the sample population are shown in Table 1. The average age of the participants was 32.54 years (*SD* = 7.32), with 54% over 30 years old. Of the participants, 75% were female, 59.8% were married, and more than half of the participants (59.3%) had a diploma/associate degree. Most participants (72.1%) had very good or good health. Participants’ average work experience was 9.30 years (SD = 7.05), with 67.1% having less than 10 years of work experience. Of these participants, 75.2% worked in rotating shifts at the time of this study. The average PSS-10 score was 17.12 (SD = 4.30) with the majority of participants (79.5%) experiencing a moderate stress level, followed by a low level (19.3%) and high level (1.2%) of stress. The average scores of PCS and MCS of SF-12 were 62.76 (SD = 22.51) and 59.07 (SD = 17.02), respectively.

**Table 1:** Participants’ demographic information (*n* = 420)

Variables	Mean (SD)	Min – Max	n (%)
Age (years)	32.54 (7.32)	22–60	
≤ 30			193 (46.0)
> 30			227 (54.0)
Gender			
Male			105 (25.0)
Female			315 (75.0)
Marital status			
Single/divorced/widowed			169 (40.2)
Married			251 (59.8)
Education level			
Diploma/Associate			249 (59.3)
Bachelor’s/Graduate			171 (40.7)
Health condition			
Very good/Good			303 (72.1)
Fair/bad/very bad			117 (27.9)
Work experience (years)	9.30 (7.05)	1–38	
≤ 10			282 (67.1)
> 10			138 (32.9)
Shift work			
No			104 (24.8)
Yes			316 (75.2)
Perceived stress (PSS-10)	17.12 (4.30)	3–31	
Low			81 (19.3)
Moderate			334 (79.5)
High			5 (1.2)
Quality of life (SF-12)			
PCS	62.76 ± 22.51	4.18–100	
MCS	59.07 ± 17.02	10.0–96.7	

*SD* standard deviation, *n* frequency, *PSS-10* Perceived Stress Scale, *SF-12* Short form, *PCS* Physical component domain, *MCS* Mental component domain

### Characteristics of sleep disturbances among participants

The average GSDS score was 41.10 (SD = 19.48), with 197 (46.9%) participants scoring ≥43, indicating sleep disturbances. Regarding the seven dimensions of the GSDS, the most common complaint was that participants were waking up too early from their sleep (64%). Other complaints included poor quality of sleep (56.9%), waking up during sleep (51.2%), and a lack of sleep quantity (36.7%). Problems with falling asleep, as well as fatigue and alertness while at work, were both reported (35.2%). Only 4% of participants admitted to using substances to induce sleep (Table 2).

**Table 2:** Characteristics of sleep disturbance among participants (*n* = 420)

Sleep disturbances	Mean (SD)	Min – Max	n (%)
*Overall score*	41.10 **(**19.48)	0–126	197 (46.9)
Problems initiating sleep	2.09 **(**1.83)	0–7	148 (35.2)
Waking up during sleep	2.88 **(**2.03)	0–7	215 (51.2)
Waking too early from sleep	3.79 (2.53)	0–7	269 (64.0)
Quality of sleep	2.81 (1.52)	0–6.6	239 (56.9)
Quantity of sleep	2.14 (1.49)	0–7	154 (36.7)
Fatigue and alertness at work	2.50 (1.36)	0–7	148 (35.2)
Substances to induce sleep	.35 (1.00)	0–7	17 (4.0)

*SD* standard deviation, *n* frequency

### Associated factors with sleep disturbances

Statistically significant differences in sleep disturbance were found for age (*χ*^*2*^ *= 4.223, p* = .05), self-rated health condition (*χ*^*2*^ *= 10.878, p* = .001), stress (T = − 9.98, *p* < .001), PCS (T = 6.483, *p* < .001), and MCS (T = 6.19, *p* < .001; Table 3). This indicates that nurses aged 30 years or less, who reported fair/bad/very bad health conditions, high perceived stress, and poor QoL, experience more sleep disturbance. However, the relationship between sleep disturbance and gender, marital status, education level, work experience, and shift work was not significant (*p* > .05).

**Table 3:** Association between demographic factors and sleep disturbances (*n* = 420)

Variables	Participants with/without sleep disturbances					
	Without	With	Without	With	*a/b-value*	*p*
	Mean (SD)		n (%)			
Age (years)					4.223^a^	.05
≤ 30			92 (47.7)	101 (52.3)		
> 30			131 (57.7)	96 (42.3)		
Gender					.156^a^	.735
Male			54 (51.4)	51 (48.6)		
Female			169 (53.7)	146 (46.3)		
Marital status					.119^a^	.765
Single/divorced/widowed			88 (52.1)	81 (47.9)		
Married			135 (53.8)	116 (46.2)		
Education level					.127^a^	.766
Diploma/Associate			134 (53.8)	115 (46.2)		
Bachelor’s/Graduate			89 (52.0)	82 (48.0)		
Self-rated health condition					10.878^a^	.001
Very good/Good			176 (58.1)	127 (51.9)		
Fair/bad/very bad			47 (40.2)	70 (59.8)		
Work experience (years)					2.588^a^	.119
≤ 10			142 (50.4)	140 (49.6)		
> 10			81 (58.7)	57 (41.3)		
Shift work					1.173^a^	.309
No			60 (57.7)	44 (42.3)		
Yes			163 (51.6)	153 (48.4)		
Perceived stress (PSS-10)	15.37 (4.25)	19.11 (3.41)			−9.98^b^	< .001
Quality of life (SF-12)						
PCS	69.19 (20.21)	55.48 (22.81)			6.483^b^	< .001
MCS	63.70 (16.01)	53.83 (16.65)			6.19^b^	< .001

*SD* standard deviation, *PSS-10* Perceived Stress Scale, *SF-12* Short form, *PCS* Physical component domain, *MCS* Mental component domain

^a^Results of chi-square test (χ^2^)

^b^Results of the t-test

The variables showed a statistical significance in the univariate analysis. Demographic characteristics were considered as the independent variable, and sleep disturbance was considered as a dependent variable. Table 4 reports the ORs and 95% CI from the multiple logistic regression analysis of the relationships between sleep disturbance and single-factor analysis. Sleep disturbances were independently associated with nurses’ perceived stress and QoL (poor PCS). In other words, nurses with high stress were significantly more likely to suffer from sleep disturbance than others (OR = 1.269 [95% CI = 1.185–1.360], *p* < .001). In addition, with regard to QoL, nurses with poor physical health status were more likely to experience sleep disturbances compare with others (OR = .98 [95% CI = .968–.991], *p* < .001).

**Table 4:** The multiple logistic regression analysis (*n* = 420)

Variable and assignment	β	S.E	Wald	*p*	OR (95% CI)
Constant	−2.454	.980	6.265	.012	
Age (years)					
≤ 30	Reference				
> 30	−.219	.227	.931	.334	.803 (.514–1.254)
Self-rated health condition					
Very good/Good	Reference				
Fair/bad/very bad	.338	.254	1.77	.183	1.403 (.852–2.308)
Perceived stress (PSS-10)	.238	.035	46.185	< .001	1.269 (1.185–1.360)
Quality of life (SF-12)					
PCS	−.021	.006	12.293	< .001	.98 (.968–.991)
MCS	−.010	.008	1.813	.178	.99 (.975–1.005)

*CI* Confidence interval, *S.E.* Standard Error, *PSS-10* Perceived Stress Scale, *SF-12* Short form, *PCS* Physical component domain, *MCS* Mental component domain

## Discussion

This study examined a previously unstudied population, providing evidence of sleep disturbance and its associated factors among Vietnamese staff nurses. Overall, participants experienced a high rate of sleep disturbance. Moreover, sleep disturbance among nurses was significantly associated with high stress and poor physical health. Our findings contribute to the existing knowledge of sleep disturbance among nurses and help enhance nurses’ work quality and well-being. Differences exist in healthcare system setups, as well as differences in cultural and health-related habits across countries worldwide [10, 17]. Therefore, it is useful to refer to the prevalence of sleep disturbance among nurses in specific countries to highlight this issue and help reduce it. This study is significant as it provides evidence specifically in the Vietnamese context.

The study results showed that nearly half (46.9%) of participants reported sleep disturbance (GSDS ≥43), which was higher than the general population [36]. This finding indicates that more attention should be paid to sleep disturbances among nursing staff in Vietnam. This is consistent with a previous study that examined 155 nurses in China and found that 46% of nurses had poor QoS [37]. However, our findings are lower than those of several previous studies [8, 9, 13, 16–18, 20, 21], and higher than others [5, 10]. For example, Almhdawi et el. studied 597 nurses in Jordan and found that 68% experienced QoL shortages [17]. Park et al. conducted a study on 188 nurses in Korea and reported that 79.8% experienced poor QoS [18]. Similarly, a study including 2003 nurses in China showed that 42.9% experienced poor QoS [5]. The differences among reported sleep disturbance in different countries may be the result of differing working and living conditions [10]. For example, nursing staffs are typical responses for twenty to thirty patients in Vietnam [26], whereas the number is ten to eleven in Spain, nine to ten in Germany, and four to five in Sweden [38]. Comparatively lower rates found in our study may be attributed to the fact that participants were nurses working in different positions (nurse managers, general nurses who directly take care of patients, and administrative nurses). Moreover, differences among the measurement instruments may also contribute to this discrepancy. Thus, further studies are needed to obtain more evidence on the prevalence of sleep disturbances among nurses in Vietnam. Hospital staff nurses in Vietnam have to receive and care for multiple patients at the same time, with many having critical and severe diseases and requiring major surgery. To take good care of patients, nurses need to regularly update their professional knowledge hierarchy, which can increase pressure on nurses. In addition, nurses have to be on duty at night, which affects QoS. Therefore, continuing attention towards sleep disturbances among nurses is an important and urgent issue for nursing educators and administrators in Vietnam.

In this study, age was found to be significantly associated with sleep disturbance—participants aged 30 years or younger had higher rates of sleep disturbances than those aged over 30 years. This finding accords with the findings of a previous study including 152 nurses in Turkey [20], which showed that nurses in the 25–30 years age group reported higher rates of sleep disturbances than other age groups. Chung et al. studied 137 nurses and found that older nurses experienced a significantly reduced risk of sleep disturbance [39]. However, this finding is inconsistent with previous studies [20, 24]. For example, Tarhan et al. found that older age had a negative on sleep quality [20]. Differences in health systems, cultures, and health associated with habituation could account for this discrepancy. Indeed, Vietnamese young staff nurses often work in specific care departments, such as the intensive care unit, emergency department, or post-operative department with high work pressure and usually work in rotational shifts. Moreover, nurses aged 30 or under are of childbearing age. Therefore, besides working at the office, they also have to take care of their families, including children and parents. Furthermore, sometimes they are the main income source for their families, making financial pressure also affect their sleep. To reduce sleep disturbance among staff nurses, it is recommended that nurse managers give more attention and support strategies to young nurses working in critical care units.

In this study, participants with fair/bad/very bad health conditions experienced higher rates of sleep disturbance than participants who self-rated their health as good. This is consistent with the findings of a study of 597 nurses in Jordan [17], which reported that poor self-evaluation of health was significantly associated with sleep disturbance. Health status has been shown to affect nurses’ critical thinking ability, thereby affecting work productivity, job performance, and quality of care [40, 41]. This might increase potential work pressure for nurses, further affecting their sleep. Therefore, the causal effects between health status and sleep disturbances require further investigation.

This study also demonstrated that stress had a significant effect on sleep disturbance—higher stress perception scores indicate higher rates of sleep disturbance, consistent with previous studies [17, 28, 29]. Young nurses are at an increased risk of depression. It is speculated that this might be due to less work experience leading to reduced confidence levels. High workload, stressful clinical situations, low income, and family responsibilities can also cause stress [23]. Moreover, high stress increases fatigue, leading to an increased need for rest. If this need is not compensated, it might be detrimental to health, such as the increased prevalence of sleep disorders among nurses [16]. Therefore, reducing stress is an issue that needs attention as it can be used as a strategy to decrease rates of sleep disturbance among staff nurses.

Consistent with previous findings, QoL was significantly associated with sleep disturbances in our study [9–11, 36, 42]. This indicated that the better the QoL, the lower the sleep disturbance. However, only the physical health component was conserved in the final model of the multifactor logistic regression analysis for sleep disturbances. Poor physical health was associated with sleep disturbance among staff nurses. Moreover, these variables may interact with each other. Specifically, more sleep disturbances decrease physical health, and better physical health enhances QoS. This finding indicated that the pathway mediating between sleep disturbances and QoL was more likely to be physical rather than psychological. However, this study did not determine a causal association between sleep disturbance and QoL. Therefore, further interventions to improve physical health and identify the causal association between sleep disturbance and QoL are strongly recommended.

Although the association between shift work and sleep disturbances was not found in the current study, most studies have demonstrated that shift work interferes with QoS and the occurrence of sleep disturbance among nurses. In other words, day-shift nurses have a better QoS than night-shift nurses [15, 16, 20, 21, 23, 25, 37]. Therefore, nursing managers need to participate actively and strategically to decrease this negative impact.

The current study also failed to find an association between sleep disturbances and gender, marital status, education level, and work experience of nurses. This is consistent with the findings of some previous studies [21, 25, 37]. In contrast, these findings are inconsistent with those of several other studies. For example, evidence suggests that female nurses experience sleep disturbances more easily than male nurses [2, 5, 9], married nurses experience higher rates of sleep disturbance compared with unmarried nurses [15], nurses with lower education levels have a much higher risk of experiencing sleep disturbance [8], nurses with 10 years of work experience have a higher risk of sleep disturbances than nurses with 5–10 years [5]. The differences in these conclusions may be related to the sample size and differences in work environments and cultures. Therefore, further studies are strongly recommended.

This study has several limitations. First, the information was self-reported. Therefore, recall and supporting bias could have occurred during the data collection process. To minimize bias, objective measurements should be conducted. Second, the association findings from this study may not be causal owing to the study’s cross-sectional design. A longitudinal design should be conducted to clarify the causality between sleep disturbances and its associated factors. Third, the findings of this study have limited generalizability because the sample included nurses from only three hospitals located in the southwestern region of Vietnam using the convenient sampling method. Therefore, further studies using nationwide systematic sampling and international comparisons are highly recommended. Finally, participants’ emotional health (e.g., anxiety, depression, fatigue, stress, etc.) and family-related factors (e.g., childcare, financial issues, etc.) were not explored in association with sleep disturbances in our study. Thus, the relationship between these factors and sleep disturbance in staff nurses should be evaluated in future studies.

## Conclusion

The findings from the current study add to existing evidence suggesting that the prevalence of sleep disturbance among Vietnamese nursing staff is considerably high. High levels of stress and poor physical health status were found to be significant predictors. Only high stress and poor PCS were statistically significant predictors of sleep disturbance among nurses. The findings of this study provide important information for hospitals in Vietnam. Sleep disturbance is still an important health issue for staff nurses, and in-service education may be useful for decreasing sleep disturbances in this subject. Therefore, further studies using a larger sample are needed to confirm our findings.

## Data Availability

Data of this study are available from the first author on appropriate request.

## Abbreviations

GSDS: General Sleep Disturbance Scale
PSS-10: Perceived Stress Scale
MCS: Mental Component Scale
PCS: Physical Component Scale
QoL: Quality of Life
QoS: Quality of Sleep
SD: Standard Deviation
SPSS: Statistical Package for the Social Sciences
